# 

**Published:** 1896-02

**Authors:** 


### BUFFALO MEDICAL JOURNAL

Established 1845 by AUSTIN FLINT, M. D.

**EDITORS:**

THOS. LOTHROP, M. D.  WM. WARREN POTTER, M. D.

**ASSOCIATE EDITORS:**

WILLIAM C. KRAUSS, M. D.  JAMES WRIGHT PUTNAM, M. D.
JOHN A. MILLER, Ph. D.  JOHN PARMENTER, M. D.
ERNEST WENDE, M. D.  ALVIN A. HUBBELL, M. D.

EUROPEAN AGENCY, J. B. BAILLIÉRE & FILS, 19 Rue Hautefeuille, Paris.
*Subscription, if paid in advance, $2.00 ; otherwise, $2.50. Foreign subscription, $2.50.*
Copyright 1895, by William Warren Potter, M. D.
Entered at the Post Office at Buffalo, N. Y., as second-class mail matter.

A. T. BROWN PRINTING HOUSE, BUFFALO, N. Y.

Table of Contents on Page V.

